# Comparison of the anesthesia effect of ultrasound-guided middle and low interscalene brachial plexus block: a randomized, controlled, non-inferiority trial

**DOI:** 10.1186/s12871-022-01963-4

**Published:** 2023-01-03

**Authors:** Yang Zhao, Shiming Qin, Xue Yang, Chongmei Gao, Xia Yuan, Tao Li, Zhaohui Chen

**Affiliations:** 1 Department of Anesthesiology, Affiliated Hospital of North Sichuan Medical College, No. 1 The South of Maoyuan Road, Nanchong, Sichuan 637000 People’s Republic of China; 2 Department of Anesthesiology, The First Affiliated Hospital of Guangxi Medical University, No. 22 Shuangyong Road, 530021 Guangxi, People’s Republic of China; 3 Department of Anesthesiology, Third Affiliated Hospital of Chongqing Medical University (Gener Hospital), No.1 Shuanghu Branch Road, Chongqing, 401120 China; 4 Department of Radiology, Chengdu Second People’s Hospital, Chengdu, 610017 Sichuan China

**Keywords:** Block, Brachial Plexus, Methods, Anesthesia, Ultrasonography, Elbow

## Abstract

**Background:**

Ultrasound-guided low interscalene brachial plexus block (LISB) can provide satisfactory anesthesia for surgery at or below the elbow. However, the anesthesia effect of ultrasound-guided middle interscalene brachial plexus block (MISB) has not been fully investigated. We hypothesized that MISB provides a non-inferior anesthesia effect to LISB for surgery at or below the elbow.

**Methods:**

A total of 82 patients with ASA I-III (18–65 years) scheduled for elective surgery at or below the elbow were randomized to the MISB group or the LISB group equally, located 1/2 or 2/3 of the caudal distance from C6 to the clavicle. Both groups were administered 15 mL 0.5% ropivacaine at the lower part of the brachial plexus with the first injection and equivalent volume at the upper part with the second injection.

**Results:**

For the primary outcome, 92.3% in the MISB group experienced successful anesthesia compared to 94.6% in the LISB group [difference: –2.3%, 95% confidence interval (CI) –13.4% to 8.8%], exceeding the predefined non-inferiority margin -15%. For the secondary outcomes, the incidence of pleura suppression for the first injection (7.7% vs. 45.9%, *P* < 0.001) and the time to perform the block (9.9 ± 1.3 vs. 10.7 ± 1.3 min, *P* = 0.006) were significantly less in MISB compared to LISB. No significant differences were observed in the consumption of perioperative rescue analgesics, VAS score, and adverse events within the two groups.

**Conclusions:**

MISB provides a non-inferior anesthesia effect to LISB for surgery at or below the elbow.

**Trial registration:**

Chinese Clinical Trial Register (identifier: ChiCTR2100054196).

## Key points

1. We proposed the middle interscalene brachial plexus block (MISB) for the first time and assessed its efficacy for the sensory and motor block.
2. MISB provides a non-inferior anesthesia effect to low interscalene brachial plexus block (LISB) for surgery at or below the elbow.
3. MISB may be considered a valuable alternative for LISB, especially for patients with poor ultrasound images or a high risk of pneumothorax.
4. Both MISB and LISB improved the success rate of the inferior trunk compared to the classical interscalene brachial plexus block.
5. We observed the diffusion of the local anesthetics with MRI 3D STIR SPACE images.

## Introduction

The classic interscalene brachial plexus block (CISB) was performed ﻿at the C6 level in the cricoid cartilage, which has evident advantages such as not requiring moving the patient's arm or forearm in the case of trauma or abnormality, a better anesthesia effect for upper limb and shoulder surgery, and easy to learn [1, 2]. However, CISB is generally not recommended for surgery at or below the elbow because the inferior trunk (mainly ulnar nerve) is inadequately blocked ﻿up to 50% [3, 4]. Recently, some researchers have found that ultrasound-guided low interscalene brachial plexus block (LISB), located 2/3 of the caudal distance from C6 to ﻿the clavicle, significantly improved the sensory and motor block of the ulnar nerve for surgery at or below the elbow compared to CISB [5–7]. However, because it is close to the pulmonary apex, ultrasound-guided LISB still has the potential to cause pneumothorax even by a specialized anesthesiologist [8]. Therefore, we try to propose a new approach, middle interscalene brachial plexus block (MISB), located at the middle (1/2) site between C6 and the clavicle, which is far away from the pulmonary apex, unlike LISB, to reduce the incidence of pneumothorax. To the best of our knowledge, no study had been performed to compare the anesthesia effect of MISB and LISB. We performed a non-inferiority trial comparing MISB and LISB. Our primary objective was to determine whether the anesthesia effect of MISB was non-inferior to LISB. We hypothesized that MISB provides a non-inferior anesthesia effect when compared to LISB for surgery at or below the elbow, within the bounds of the predefined margin of the non-inferiority set at − 15%.

## Materials and methods

### Ethics

Ethic for this randomized, prospective, ﻿observer-blinded clinical study was approved by the Medical Ethics Committee of the third affiliated hospital of Chongqing Medical University (president Fei Hao), China 2/12/2021, approval number 2021/35. The study protocol was registered with the Chinese registry of clinical trials (http://www.chictr.org.cn) (ChiCTR2100054196; 11/11/2021) and conducted in accordance with the Helsinki Declaration-2013. This study adhered to the CONSORT guidelines and was conducted from January 7 to May 11, 2022, at the third affiliated hospital of Chongqing Medical University [9]. Informed written consent was obtained from the eligible participants a night before surgery in the ward.

### Patients

Inclusion criteria were patients aged 18 to 65 years; American Society of Anesthesiologists classification (ASA) I–III; ability to express pain; and patients undergoing elective surgery at or below the elbow. Exclusion criteria were patients who ﻿had neck tissue abnormality, infection, or edema; impaired neurological function; coagulation disorder; a history of an allergic reaction to local anesthetics or opioids; weighing < 40 kg (to reduce the risk of anesthetic toxicity) or > 100 kg (to reduce the difficulty of performing ISB); psychiatric dysfunction and patients who refused to sign informed consent.

### ﻿Randomization and blinding

On the day of surgery, consented patients were randomly assigned to the LISB or MISB group (1:1) using SPSS 25.0 software (Statistical Program for Social Sciences, SPSS Inc., Chicago, Illinois, USA) by C.-M.G. who was not involved in the study. Allocation anonymity was ensured by enclosing assignments in sealed, opaque, and sequentially numbered envelopes opened by X.Y. Then, X.Y. prepared 0.5% ropivacaine (30 mL) for patients during the study period only upon their arrival in the operation room [10]. After Z.-H.C. performed ultrasound-guided LISB or MISB, a senior anesthesiologist who did not participate in any other related process according to the allocation, an opaque gauze was used to cover the injection site to blind the observer. Another anesthesiologist S.-M. Q., blinded to the allocation, evaluated the sensory and motor block scale and administered anesthetic drugs when appropriate. The allocation was blinded for patients, surgeons, the anesthesiologist (S.-M. Q.), and observers until the end of the study.

### Intervention technique

**Fig. 1:**
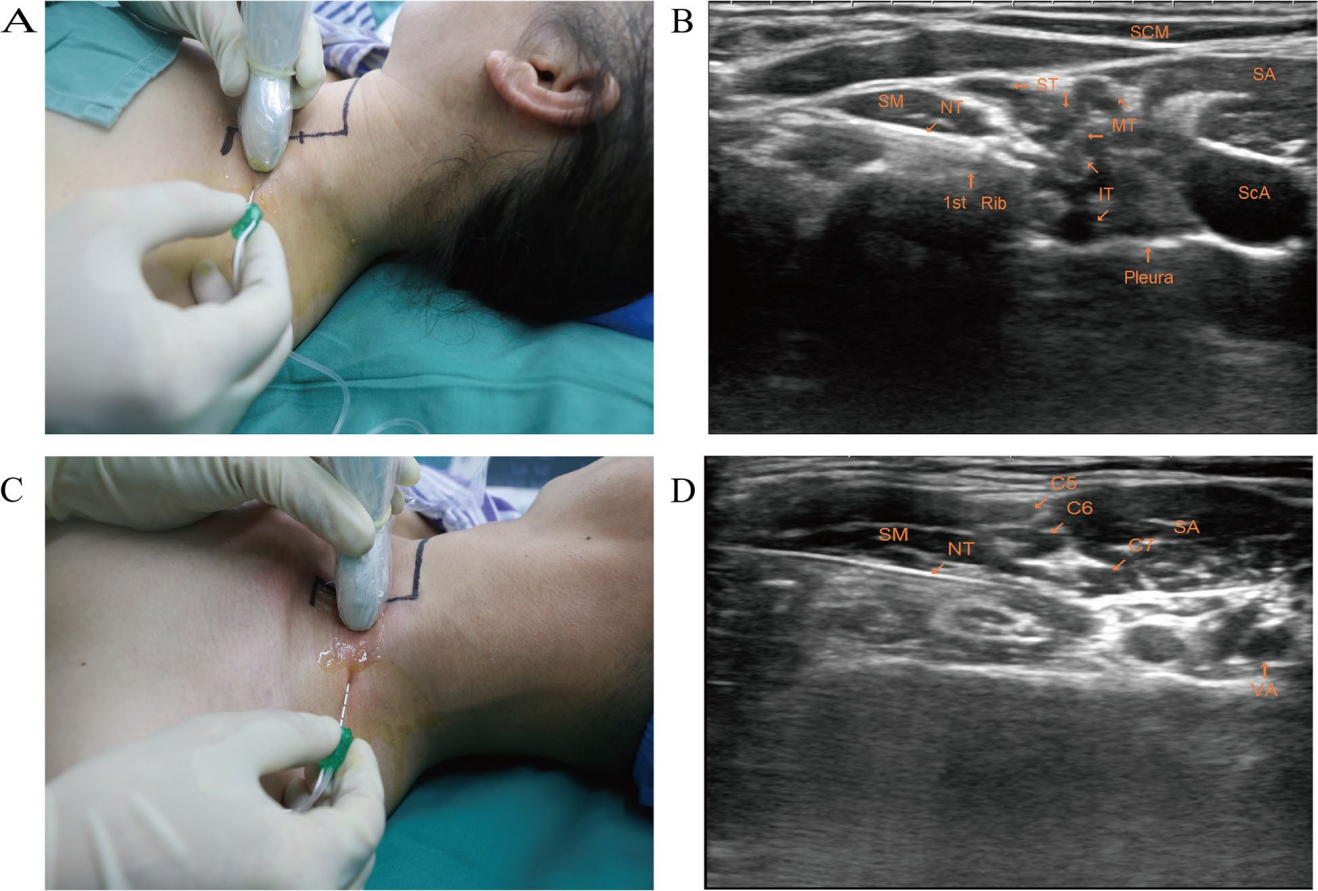
Location and an ultrasound image of LISB and MISB. Note: **A** and **C**: The patient in LISB group (**A**) or MISB group (**C**) was kept in the supine position with their heads facing away from the side of the block, and the transducer was placed on the interscalene groove, located 2/3 (A) or 1/2 (**C**) of the caudal distance from C6 to the clavicle. **B **and **D**: Ultrasonic image of LISB (**B**) or MISB (**D**) shows the first needle trajectory in-plane and advanced toward the lower part of the brachial plexus**.** I.T. = inferior trunk; MISB = middle interscalene brachial plexus block; M.T., middle trunk; LISB = low interscalene brachial plexus block; N.T., needle trajectory; S.A., scalenus anterior muscle; ScA, subclavian artery; SCM, sternocleidomastoid muscle; S.M., scalenus medius muscle; S.T., superior trunk; V.A., vertebral artery

Venous access was established after arrival in the operating room without any premedication before anesthesia. During the same time, the vital signs of the patients were monitored every three minutes using a non-invasive blood pressure parameter, pulse oximeter, and electrocardiogram. Patients were kept supine with their heads facing away from the block side. All blocks were performed by the senior anesthesiologist (Z.-H.C.), who had performed over 200 ultrasound-guided peripheral nerve blocks. After sterilizing the region with povidone-iodine, 2% lidocaine (1 mL) was injected for local site subcutaneous infiltration. An 11 MHz linear probe (HITACHI ALOKA ARIETTA, America) was also wrapped with a sterilized plastic cover. The probe was located at a caudal 2/3 distance from C6 to ﻿the clavicle in the LISB group (Fig. 1 A and B). The probe was placed in the middle (1/2) between C6 and the clavicle in the MISB group (Fig. 1 C and D). The middle and anterior scalene muscles should be recognized clearly for all participants, with the transducer held in the corresponding location according to the allocation. Double injections were performed because they may facilitate ﻿better distal sensory and motor blocks in the brachial plexus block [11]. After the nerve roots of the brachial plexus were seen as several hypoechoic ovoid structures in the interscalene groove, a sterilized 22G 50 mm stimulating needle (B. Braun Melsungen AG, Germany) was inserted into the plane and advanced toward the lower part of the brachial plexus. The first administration of half volume (15 mL 0.5% ropivacaine, 75 mg) was performed upon any muscle contraction (pectoralis, deltoid, biceps brachii, triceps brachii fingers) confirmed with the help of a nerve stimulator set at 0.4 mA current with 2 Hz frequency. Subsequently, ﻿the remaining volume (15 mL 0.5% ropivacaine, 75 mg) was given by shifting the needle tip toward the upper part of the brachial plexus regardless of any muscle contraction. The contraction of each muscle was recorded during the double injection process, and the suppression of pleural membrane under ultrasound-guided was recorded while administering the first injection.

### Perioperative analgesia and management

Data on age, sex, weight, height, BMI, surgical site, and ASA classification were collected. ﻿The anesthesia effects were defined as below: "success ", the surgery was performed under the block with or without additional sufentanil and propofol intravenously administered at a maximum usage of 10 ug or 2 mg/kg, respectively; "failure", reverted to trachea intubation or general anesthesia due to inadequate analgesia or other adverse invents. After the block procedure, a blinded anesthesiologist (S.m.Q.) performed sensory, and motor blockade evaluation, unaware of the allocation at 5 min, 15 min since the needle was withdrawn from the patient and the end of the surgery. The extent of sensory block and loss of cold sensation were assessed using an ice cube in areas supplied by six nerves according to a 3-point scale (0 = normal cold sensation, no block; 1 = partial block of cold sensation, partial block, and 2 = no cold sensation, complete anesthesia): ulnar (fifth finger), musculocutaneous (lateral forearm), radial (dorsal skin between the thumb and second finger), median (a palmar aspect of the second finger), medial cutaneous nerve of the forearm (medial side of the forearm), and axillary (stump shoulder). The extent of the motor block was assessed by specific motor activity: ulnar (fourth and fifth finger flexion), musculocutaneous (elbow flexion), radial (finger extension), median (wrist flexion), and axillary (shoulder abduction) (0 = normal, 1 = partial paralysis, 2 = complete paralysis) [2, 11]. ﻿These were compared with those of the contralateral side. All patients received ketorolac 30 mg intravenously for postoperative analgesia every 6 h. If the patient complained on the Visual Analog Scale (VAS, 0 = no pain; 10 = worst pain imaginable) at resting over 3 (moderate pain), a muscular injection of rescue tramadol (50 mg) was offered [12]. The patients were followed up until discharge.

### Magnetic resonance imaging of the brachial plexus

﻿Magnetic resonance imaging was performed immediately after surgery on a 1.5 T magnet (Siemens Aera MR, Germany) to observe the diffusion of the local anesthetics. MRI examination consents were obtained from the two patients of the corresponding group. The patient in the supine and head advanced position was placed in ﻿the head, neck, and spine matrix coils. The magnetic field center was scheduled for the C6 level and scanned sequences included the transverse T1WI, T2WI, and coronal T2WI. ﻿M.R. neurography was performed using 3D short-tau inversion recovery and SPACE imaging (3D STIR SPACE), TR 3000 ms, TE 248 ms, TI 180 ms, 448 × 448 mm field-of-view (FOV), ﻿448 × 448 resolution, 1 mm slice thickness. The original images were transmitted to the Siemens workstation to process maximum intensity projection (MIP), multiplanar reconstructions (MPR) and image analysis.

### Outcome measures

The primary outcome was the anesthesia success rate with a non-inferiority test. Secondary outcomes included: incidence of suppression of the pleura when administering the first injection; the sensory and motor blockade scores of all branch nerves at 5 min, 15 min, and the end of surgery; evaluation of the motor response of double injections; ﻿the time to perform ISB, which was defined as the time from the start of initial scanning to the withdrawal of the needle; intraoperative dosage of propofol and sufentanil; the cumulative consumption of tramadol within 24 h after surgery; resting and moving VAS scores at 2, 4, 6, 12 and 24 h after surgery; duration of surgery defined as the time from the start of surgical incision to the end of the last stitch; time to readiness for surgery defined as the time from withdrawal of the needle to the start of surgical incision would also be recorded. Simultaneously, ISB-related side effects such as nausea, vomiting, Horner Syndrome, dyspnea, hoarseness, pneumothorax, and anesthetic toxicity were recorded.

### Sample size and statistical analysis

This study was designed to compare the non-inferiority of the anesthesia success rate in MISB and LISB undergoing surgery at or below the elbow. Previous studies reported that the success rate of low interscalene brachial plexus block anesthesia for surgery at or near the elbow was approximately 95% when opiates and midazolam were used in advance, and a low dose of propofol was used for continuous sedation [7]. As the anesthesia success rate 80% was considered enough for the surgery at or below the elbow under brachial plexus block, so we set a non-inferiority margin (minimum clinically meaningful difference) of the success block rate as 15% [6]. We consider that the success rate of anesthesia between the two groups is similar; using these estimates, a sample size of 33 was required for each group to achieve 80% power to establish non-inferiority of ultrasound-guided MISB with an error of 0.025 (one side). Considering 20% dropout, we recruited 82 patients (41 per group).

The primary endpoint was assessed by the non-inferiority test for the difference between two proportions (the null hypothesis that the difference in the anesthesia success rate was greater than or equal to 15% vs. the alternative hypothesis that the difference was less than 15%). The 95% confidence interval for the difference in anesthesia success rate between MISB and LISB was calculated. If the lower 95% confidence limit was above –15%, the anesthesia effect of MISB was deemed to be non-inferior to LISB.

For other outcomes, after confirming the normality of distribution using the Shapiro–Wilk test, continuous variables were compared using the t-test or Mann–Whitney test. As appropriate, continuous variables were presented as means ± SDs or median (interquartile range). Categorical variables were compared by the chi-square or Fisher exact test and presented as numbers and percentages. Data analysis was performed using SPSS software (Statistical Program for Social Sciences, SPSS Inc., Chicago, Illinois, USA). with a two-tailed *P*-value < 0.05.

## Results

### Patient characteristics

**Fig. 2:**
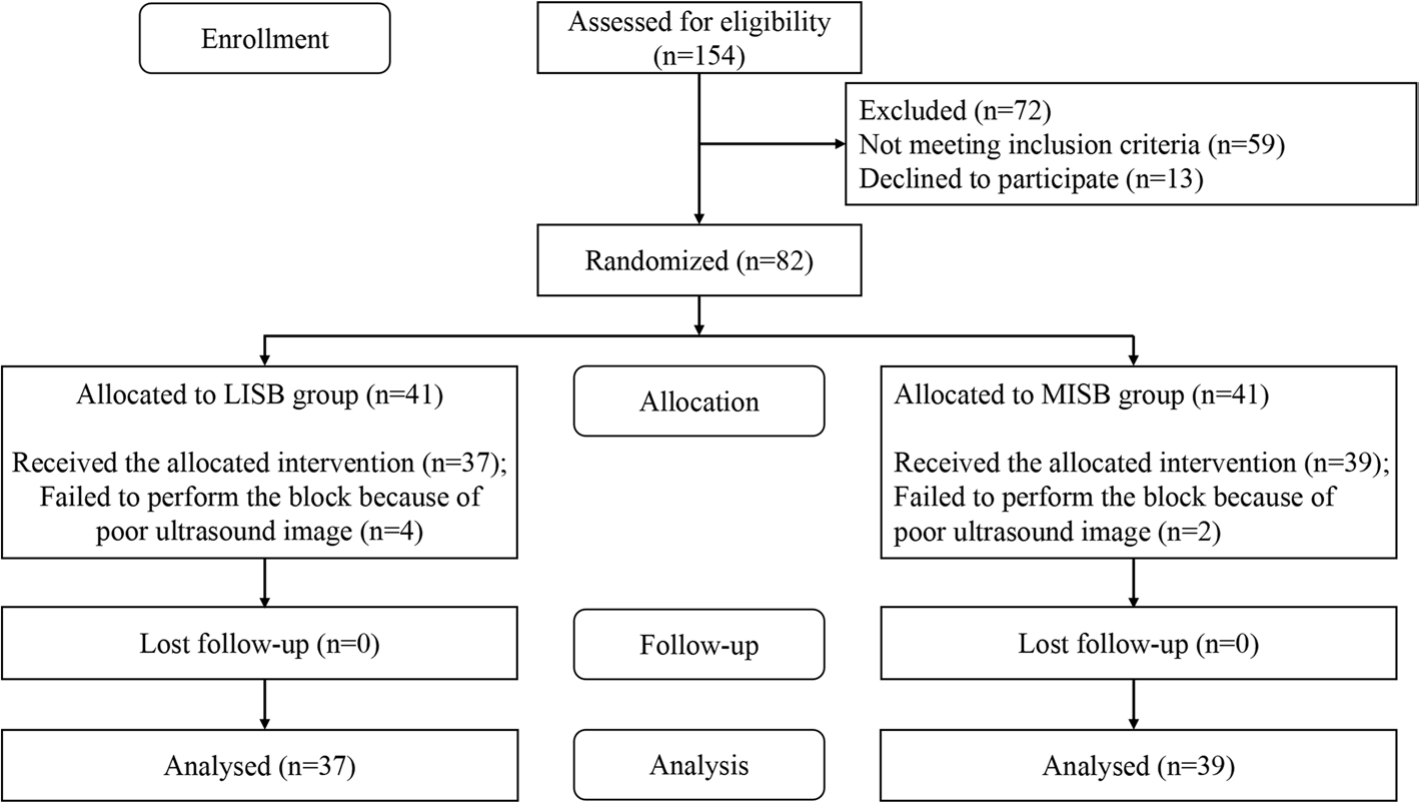
Consort flow study diagram. Note: MISB = middle interscalene brachial plexus block; LISB = low interscalene brachial plexus block

One hundred fifty-four consecutive patients were assessed for eligibility between January 2022 and May 2022. Of these, 59 patients did not meet the inclusion criteria and 13 refused to participate. The remaining 82 patients were randomized into the LISB (*n* = 41) and MISB (*n* = 41). Four patients from the LISB group and two from the MISB group were excluded because of poor ultrasound images. No patients were lost during follow-up. Finally, thirty-seven patients from the LISB group and thirty-nine from the MISB group were analyzed (Fig. 2). There were no significant differences between the two groups concerning demographic characteristics, ASA classification, and surgical site (Table 1).

**Table 1:** Baseline characteristics of study patients

﻿Characteristic	LISB Group (*n* = 37)	MISB Group (*n* = 39)	*P* value
Age (yr)	42.0 ± 12.3	43.2 ± 12.3	0.664
Sex (male/female)	27/10(73%/ 27%)	28/11(71.8%/ 28.2%)	0.909
Height (cm)	165.5 ± 8.5	166.0 ± 7.2	0.776
Weight (kg)	64.1 ± 10.8	63.7 ± 9.7	0.852
BMI (kg/m2)	23.4 ± 3.3	23.0 ± 2.8	0.646
﻿ASA physical status			0.388
I	21(56.8%)	28(71.8%)	
II	12(32.4%)	8(20.5%)	
III	4(10.8%)	3(7.7%)	
Types of surgery			0.896
elbow	2(5.4%)	2(5.1%)	
forearm	8(21.6%)	6(15.4%)	
hand	25(67.6%)	28(71.8%)	
﻿ wrist	2(5.4%)	3(7.7%)	

Note: Data are presented as mean ± SD, or number (%). *ASA*   American Society of Anesthesiologists, *BMI*   Body mass index, *MISB*   Middle interscalene brachial plexus block, *LISB  * Low interscalene brachial plexus block. Comparisons were performed using the Student's t-test, Chi-square, or Fisher exact test

### Primary outcome

**Fig. 3:**
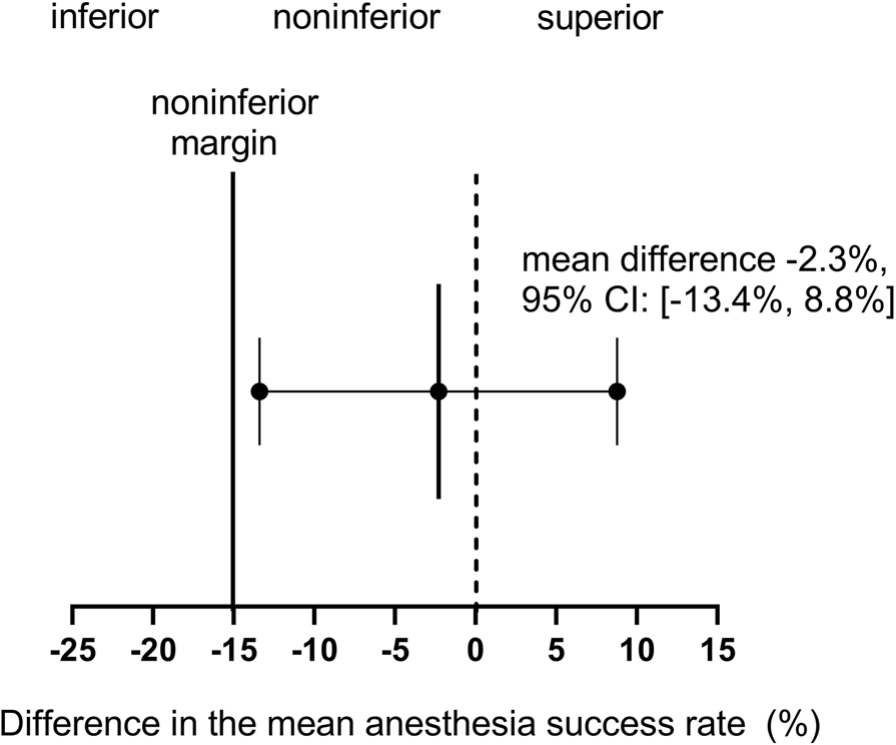
Mean difference in the anesthesia success rate. A non-inferiority test for the difference between two proportions was performed to determine the differences between MISB and LISB. The black line at the mean difference − 15% indicates the non-inferiority margin. The region to the right of the black line indicates non-inferiority, the region to the left of the black line indicates inferiority, and the region to the right of the dotted line indicated superiority. CI, confidence interval

The anesthesia success rate was 92.3% in the MISB group and 94.6% in the LISB group. The mean difference in the anesthesia success rate between the two groups was –2.3%, with a 95% confidence interval (CI) of –13.4% to 8.8%. With the non-inferiority margin set at − 15%, MISB was confirmed to provide a non-inferior anesthesia effect compared to LISB. (Fig. 3).

### Secondary outcomes

For the incidences of suppression of the pleura when administering the first volume of local anesthetics, 3 patients (7.7%) in the MISB group showed significantly less than 17 patients (45.9%) in the LISB group (*P* < 0.001). Also, the time to perform the block was significantly less in the MISB group compared to the LISB group (9.9 ± 1.3 vs. 10.7 ± 1.3 min, *P* = 0.006). However, there were no significant differences in time to readiness for surgery, duration of surgery, intraoperative use of sufentanil and propofol, cumulative tramadol consumption within 24 h after surgery, resting and moving VAS scores at all time points of follow-up (all *P* > 0.05). For adverse events, only one patient in the MISB group suffered from hoarseness, but no significant differences were found between the two groups (Table 2).

**Table 2:** Comparison of the primary and secondary outcomes between the two groups

﻿Characteristic	LISB Group (*n* = 37)	MISB Group (*n* = 39)	*P* value
Anesthesia effects (success/ failure)	35/2 (94.6%/ 5.4%)	36/3 (92.3%/ 7.7%)	0.687
Pleural membrane suppression when administering the first injection	17(45.9%)	3(7.7%)	< 0.001
Time to perform the ISB block (min)	10.7 ± 1.3	9.9 ± 1.3	0.006
Time to readiness for surgery (min)	29 [13, 14]	29 [13, 14]	0.616
Duration of surgery (min)	78[62, 110]	65 [56, 82]	0.069
Intraoperative sufentanil usage (ug)	0 [0, 0]	0 [0, 0]	0.595
Intraoperative propofol usage (mg)	0 [0, 0]	0 [0, 0]	0.781
Cumulative consumption of tramadol within 24 h after surgery (mg)	0 [0,50]	0 [0, 50]	0.908
Adverse effects			
Nausea	0	0	/
Vomiting	0	0	/
Horner Syndrome	0	0	/
Dyspnea	0	0	/
Hoarseness	0	1(2.6%)	0.327
Pneumothorax	0	0	/
Toxicity of local anesthetics	0	0	/
VAS scores at rest			
2 h	0	0	/
4 h	0	0	/
6 h	0	0	/
12 h	0.3 ± 0.8	0.3 ± 1.0	0.854
24 h	2.5 ± 0.8	2.3 ± 0.8	0.400
VAS scores at movement			
2 h	0	0	/
4 h	0	0	/
6 h	0	0	/
12 h	0.5 ± 1.6	0.4 ± 1.4	0.710
24 h	5.1 ± 1.2	5.0 ± 1.1	0.765

Note: Data are presented as mean ± SD, median (range), or number (%). *ISB*   Interscalene brachial plexus block, *MISB*   Middle interscalene brachial plexus block, *LISB*   Low interscalene brachial plexus block. Comparisons were performed using the Student's t-test, Mann–Whitney U test, Chi-square test, or Fisher exact test

In addition to an inferior sensory blockade for the ulnar and medial cutaneous nerve of the forearm nerves at 5 min and 15 min evaluations in the MISB group compared to LISB, similar extents of sensory and motor block were observed for all branch nerves at different times in the two groups (Table 3).

**Table 3:** Comparison of the sensory and motor blockade scores at different time points between the two groups

Characteristic	LISB Group (*n* = 37)	MISB Group (*n* = 39)	*P* value
Sensory block (0/ 1/ 2)			
ulnar nerve at 5 min	3/ 29/ 5 (8.1%/ 78.4%/ 13.5%)	11/ 27/ 1 (28.2%/ 69.2%/ 2.6%)	0.020
ulnar nerve at 15 min	0/ 8/ 29 (0/ 21.6%/ 78.4%)	1/23/15 (2.6%/ 59%/ 38.5%)	0.001
ulnar nerve at the end of the surgery	0/ 0/ 37 (0/ 0/ 100%)	0/ 0/ 39 (0/ 0/ 100%)	/
musculocutaneous nerve at 5 min	4/ 25/ 8 (10.8%/ 67.6%/ 21.6%)	7/ 28/ 4 (17.9%/ 71.8%/ 10.3%)	0.321
musculocutaneous nerve at 15 min	0/ 11/ 26 (0/ 29.7%/ 70.3%)	0/ 17/ 22 (0/ 43.6%/ 56.4%)	0.211
musculocutaneous nerve at the end of the surgery	0/ 0/ 37 (0/ 0/ 100%)	0/ 0/ 39 (0/ 0/ 100%)	/
radial nerve at 5 min	3/ 29/ 5 (8.1%/ 78.4%/ 13.5%)	5/ 32/ 2 (12.8%/ 82.1%/ 5.1%)	0.381
radial nerve at 15 min	0/ 15/ 22 (0/ 40.5%/ 59.5%)	0/ 19/ 20 (0/ 48.7%/ 51.3%)	0.474
radial nerve at the end of the surgery	0/ 0/ 37 (0/ 0/ 100%)	0/ 0/ 39 (0/ 0/ 100%)	/
median nerve at 5 min	4/ 31/ 2 (10.8%/ 83.8%/ 5.4%)	7/ 31/ 1 (17.9%/ 79.5%/ 2.6%)	0.572
median nerve at 15 min	0/ 13/ 24 (0/ 35.1%/ 64.9%)	0/22/17 (0/ 56.4%/ 43.6%)	0.063
median nerve at the end of the surgery	0/ 0/ 37 (0/ 0/ 100%)	0/ 0/ 39 (0/ 0/ 100%)	/
medial cutaneous nerve of the forearm at 5 min	3/ 24/ 10 (8.1%/ 64.9%/ 27%)	11/ 25/ 3 (28.2%/ 64.1%/ 7.7%)	0.016
medial cutaneous nerve of the forearm at 15 min	0/ 8/ 29 (0/ 21.6%/ 78.4%)	1/23/15 (2.6%/ 59%/ 38.5%)	0.001
medial cutaneous nerve of the forearm at the end of the surgery	0/ 0/ 37 (0/ 0/ 100%)	0/ 0/ 39 (0/ 0/ 100%)	/
axillary nerve at 5 min	6/ 19/ 12 (16.2%/ 51.4%/ 32.4%)	8/ 23/ 8 (20.5%/ 59%/ 20.5%)	0.493
axillary nerve at 15 min	0/ 9/ 28 (0/ 24.3%/ 75.7%)	0/ 13/ 26 (0/ 33.3%/ 66.7%)	0.387
axillary nerve at the end of the surgery	0/ 0/ 37 (0/ 0/ 100%)	0/ 0/ 39 (0/ 0/ 100%)	/
Motor block (0/ 1/ 2)			
ulnar nerve at 5 min	11/ 26/ 0 (29.7%/ 70.3%/ 0)	18/ 21/ 0 (46.2%/ 53.8%/ 0)	0.141
ulnar nerve at 15 min	4/ 27/ 6 (10.8%/73.0%/16.2%)	10/ 27/ 2 (25.6%/ 69.2%/ 5.1%)	0.096
ulnar nerve at the end of the surgery	0/ 0/ 37 (0/ 0/ 100%)	0/ 2/ 37 (0/ 5.1%/ 94.9%)	0.497
musculocutaneous nerve at 5 min	5/31/1 (13.5%/83.8%/2.7%)	11/27/1 (28.2%/69.2%/2.6%)	0.282
musculocutaneous nerve at 15 min	0/29/8 (0/78.4%/21.6%)	0/29/10 (0/74.4%/25.6%)	0.680
musculocutaneous nerve at the end of the surgery	0/0/37 (0/ 0/ 100%)	0/0/39 (0/ 0/ 100%)	/
radial nerve at 5 min	10/27/0 (27%/73%/0)	10/29/0 (25.6%/74.4%/0)	0.891
radial nerve at 15 min	4/ 30/ 3 (10.8%/ 81.1%/ 8.1%)	3/32/4 (7.7%/82.1%/10.3%)	0.861
radial nerve at the end of the surgery	0/0/37 (0/ 0/ 100%)	0/0/39 (0/ 0/ 100%)	/
median nerve at 5 min	9/28/0 (24.3%/ 75.7%/ 0)	13/26/0 (33.3%/ 66.7%/0)	0.387
median nerve at 15 min	4/ 30/ 3 (10.8%/ 81.1%/ 8.1%)	4/ 32/ 3 (10.3%/ 82.1%/ 7.7%)	0.994
median nerve at the end of the surgery	0/0/37 (0/ 0/ 100%)	0/1/38 (0/ 2.6%/ 97.4%)	1.000
axillary nerve at 5 min	8/28/1 (21.6%/75.7%/2.7%)	14/25/0 (35.9%/64.1%/0)	0.206
axillary nerve at 15 min	1/27/9 (2.7%/73%/24.3%)	2/28/9 (5.1%/71.8%/23.1%)	0.858
axillary nerve at the end of the surgery	0/0/37 (0/ 0/ 100%)	0/0/39 (0/ 0/ 100%)	/

Note: Data were presented as presented as numbers (percentages), and compared by the chi-square or Fisher exact test. 0 = normal, no cold sensation or no paralysis; 1 = partial block of cold sensation or partial paralysis; 2 = no cold sensation, complete anesthesia or complete paralysis. *MISB*   Middle interscalene brachial plexus block, *LISB*   Low interscalene brachial plexus block

**Fig. 4:**
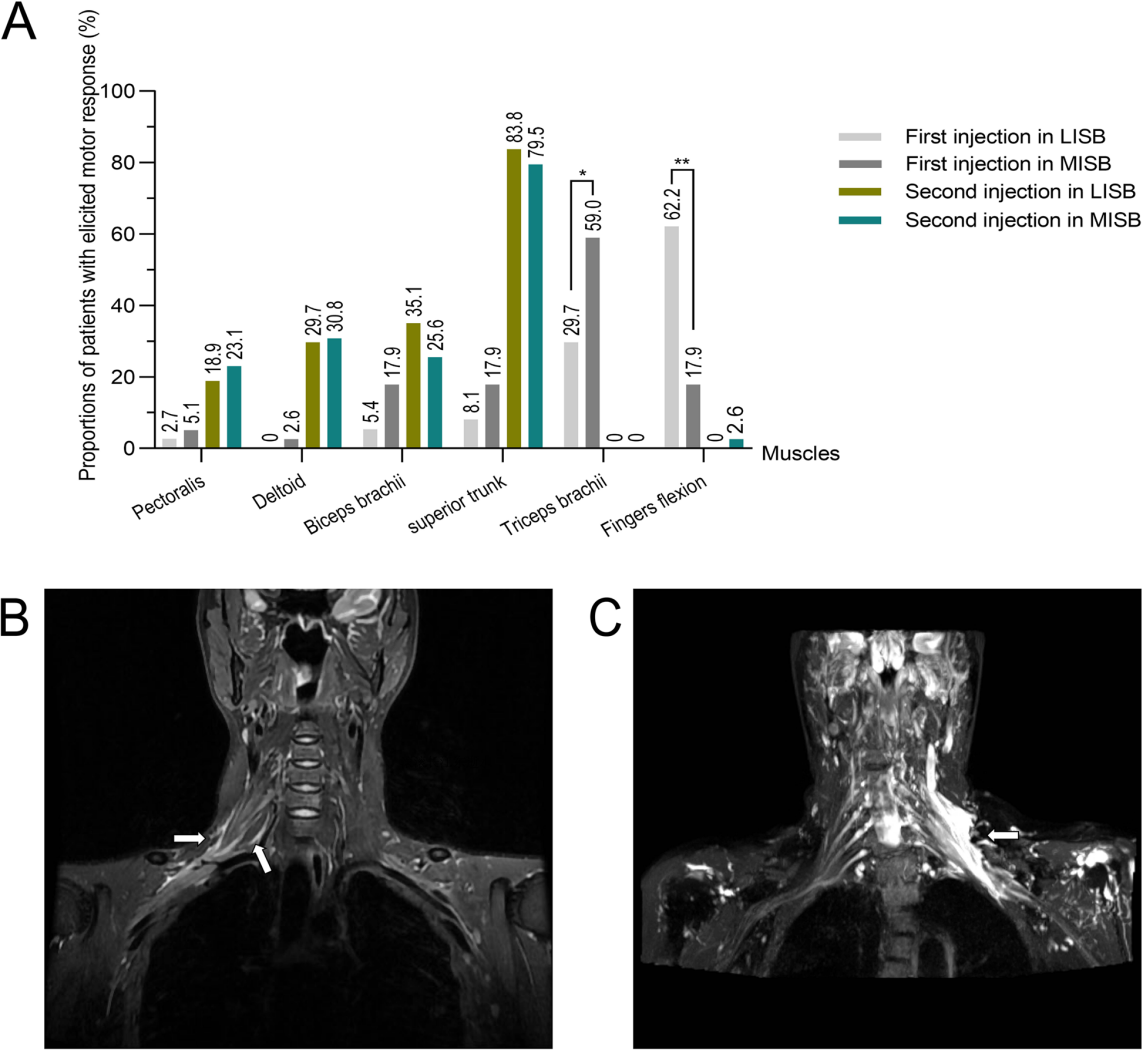
Motor response of double injection and the MRI images of ropivacaine diffusion in the two groups. Note: **A** Comparison of the proportions of the patients with elicited motor response of double-injection between the two groups. **B** MRI image of LISB group, arrow indicated hyperintense signal changes in images weighed T2 in the ventral rami T1, which extended to three trunks of the right brachial plexus. **C** MRI image of MISB group, arrow indicated hyperintense signal changes in T2-weighted images in the C5–6 ventral rami, which extended to three trunks and three cords of the left brachial plexus

At the first injection, the proportion of patients in the MISB group with fingers elicited motor response was lower (17.9% vs. 62.2%, *P* < 0.001) but higher with triceps brachii elicited motor response (59% vs. 29.7%, *P* = 0.01), compared with LISB group. However, the elicited motor response evaluations were similar between the two groups at the second injection. (Fig. 4A). After surgery, a 40-year-old male patient, 170 cm height, 75 kg weight from the LISB group, was sent for MRI examination. The sensory block scored 1, 2 for the ulnar nerve and 2, 2 for the medial cutaneous nerve of the forearm at 5 min and 15 min, respectively. Local anesthesia was observed as hyperintense signal changes in images weighed T2 in the ventral rami T1, which extended to three trunks of the right brachial plexus in the LISB group (Fig. 4B). On the contrary, an MRI of a 22-year-old male patient, 173 cm height, 58 kg weight, from the MISB group, the sensory block scored 1, 2 for the ulnar nerve and 1, 2 for the medial cutaneous nerve of the forearm at 5 min and 15 min, respectively. Local anesthetics in the MISB group were seen as hyperintense signal changes in T2-weighted images in the C5–6 ventral rami, which extended to three trunks and three cords of the left brachial plexus (Fig. 4C).

## Discussion

To date, this is the first study to compare MISB with LISB. This randomized, controlled, ﻿observer-blinded non-inferiority trial demonstrates that the anesthesia success rate of MISB is non-inferior to LISB for surgery at or below the elbow, with the margin of the non-inferiority set at − 15%.

In the scope of this study, the sensory blockade of the ulnar and medial cutaneous nerve of the forearm nerve in the MISB group was inferior to the LISB group at 5-min and 15-min evaluations but similar at the end of the surgery in both groups, indicating that the inferior trunk was blocked slower in the MISB group. Previous studies found that the onset time of complete motor block of brachial plexus was about 30 min for different approaches when administering 0.5% ropivacaine; thus, more than half of the patients in our study suffered comparable and incomplete motor block at 5 min and 15 min assessments because of inadequate onset time [2, 11, 13]. 30 ml 0.5% ropivacaine was generally recommended for interscalene brachial plexus anesthesia to achieve satisfactory sensory and motor blockage [10, 14]. Besides, a double injection technique was performed in our study due to it may facilitate ﻿better distal sensory and motor block [11, 15].MRI examinations also showed that three cords were surrounded by ropivacaine in both groups after surgery. Taking these into account, we assumed that sufficient time (30 min before surgery), enough volume, and a double injection technique might offset the disadvantages of slow sensory blockade of the inferior trunk in the MISB group, which may be the reasons why MISB produced a non-inferior anesthesia effect on LISB (92.3% vs. 94.6%, mean difference: –2.3%, 95% CI [ –13.4%, 8.8%]).

The deltoid, pectoralis, and biceps were mainly dominated by the C5 and C6 nerves, and any contraction of these muscles was considered the motor response of the superior trunk [16, 17]. Similarly to previous studies, no extension of the wrist or fingers but the contraction of the triceps brachii was observed in our study with a current set at 0.4 mA when stimulated in the ventral ramus C7, which contributed largely to the radial nerve, so the contraction of the triceps brachii was considered to be the motor response to the stimulation of the middle trunk [18, 19]. Due to the high accuracy feature, flexion of the fingers was considered the motor response elicited by the inferior trunk [20, 21]. In the present study, for the first injection, the percentage of patients in the MISB group with fingers elicited motor response was lower (17.9% vs. 62.2%, *P* < 0.001) but higher with triceps brachii elicited motor response (59% vs. 29.7%, *P* = 0.01), compared with LISB group. However, for the second injection, the motor response of the superior trunk was similar between the two groups. Thus, we assumed that MISB injection was probably performed directly around the middle and superior trunks, which eventually extended to the inferior trunk and three cords. In contrast, LISB injection was presumably performed directly around three trunks, extending to the three cords.

Similar to the previous study, superior and middle trunks can be clearly shown in both groups, while C8/T1 ventral rami could be only observed in minor patients for the LISB group [22]. For those patients without a clear image of C8/T1 ventral rami in the LISB group, we administered ropivacaine when the motor response was elicited of any trunk by titrating the needle tip to the medial of the first rib, adjacent to the subclavian artery, or under the middle trunk [3, 23]. However, the incidence of suppression of the pleura was observed more frequently in the LISB group (45.9% vs 7.7%, *P* < 0.001) at the first injection, suggesting that LISB may have greater potential to cause pneumothorax due to its closer location to the pleura than in the MISB group. Two patients from the MISB group and four from the LISB group were excluded from the study because the brachial plexus and its adjacent structures cannot be clarified clearly in ultrasound images. Even though the time to perform the block was significantly less in MISB compared to LISB (9.9 ± 1.3 vs. 10.7 ± 1.3 min, *P* = 0.006), its clinical significance was relatively limited. Thus, MISB may be a valuable alternative when encountering poor ultrasound images or a high risk of pneumothorax for LISB.

Two patients (5.4%) in the MISB group and three patients (7.7%) in the LISB group underwent trachea intubation and general anesthesia. The reasons may be the long distance for extending, the "compartment effect" of trunks or cords separated by blood vessels or muscle slip, the septum between the lateral cord and medial and posterior cords, and additional communicating branches between the components of the brachial plexus [24–29]. Most patients in the two groups had satisfied pain relief within 12 h. However, after that, the tramadol requirement gradually increased, which was consistent with previous studies in arthroscopic shoulder surgery of ISB because ropivacaine was absorbed and metabolized gradually after 12 h follow-up [30, 31]. A patient with right MISB suffered hoarseness but recovered without treatment, perhaps due to the spread of local anesthetics to the right vagus nerve, a common complication of ISB, and is safe if properly monitored [32–34]. Hypoxia, dyspnea, Horner syndrome, and local anesthetic toxicity were not observed in the patients in our study, indicating that both MISB and LISB are feasible and safe approaches for patients.

Our study had several limitations. First, the sensory and motor blockade of the nerves was not measured at the 30-min evaluation because it was inconvenient since the arm had been covered with sterile sheets and disinfected with povidone-iodine, which could interfere with the sensory blockade evaluation using an ice cube. As the anesthesia success rate was similar, we assumed that sensory and motor blockades of all nerves might not have significant differences between the two groups at the beginning of surgery (30 min). Second, the percentages of the motor response at the second injection may be underestimated due to the "short-circuit" effect of local anesthetics at the first injection. Third, theoretically, observing the local anesthetics diffusion trajectory would be more helpful if the patient's MRI examinations were carried out directly after the block was performed. However, it was unreasonable to postpone surgery since the block was performed in the operating room for the safe guarantee of the patients. Further studies are needed to perform instant MRI examinations after MISB or LISB on volunteers.

In summary, this prospective randomized trial of non-inferiority demonstrated that the MISB ﻿approach with 0.5% 30 mL of ropivacaine provided a non-inferior anesthesia effect compared to LISB for surgery at or below the elbow, within the bounds of the predefined margin of the non-inferiority set at -15%. We suggest MISB be a valuable alternative for LISB, especially for the patients with poor ultrasound images or a high risk of pneumothorax.

## Data Availability

The data used and/or analyzed during the current study are also available from the corresponding author upon reasonable request.
